# Neuroprotective Properties of a Novel Non-Thiazoledinedione Partial PPAR-****γ**** Agonist against MPTP

**DOI:** 10.1155/2013/582809

**Published:** 2013-10-02

**Authors:** Christine R. Swanson, Eric Du, Delinda A. Johnson, Jeffrey A. Johnson, Marina E. Emborg

**Affiliations:** ^1^Preclinical Parkinson's Research Program, Wisconsin National Primate Research Center, University of Wisconsin-Madison, 1220 Capitol Court, Madison, WI 53715, USA; ^2^Neuroscience Training Program, University of Wisconsin-Madison, Madison, WI 53715, USA; ^3^Department of Neurology, University of Pennsylvania Perelman School of Medicine, 3610 Hamilton Walk, Philadelphia, PA 19104, USA; ^4^Department of Epidemiology, Rollins School of Public Health, Emory University, 1518 Clifton Road, Atlanta, GA 30322, USA; ^5^School of Pharmacy, 777 Highland Avenue, University of Wisconsin-Madison, Madison, WI 53705, USA; ^6^Department of Medical Physics, 1111 Highland Avenue, University of Wisconsin-Madison, Madison, WI 53705, USA

## Abstract

Activation of the peroxisome proliferator activated receptor-gamma (PPAR)-**γ** is proposed as a neuroprotective strategy to treat neurodegenerative disorders. In this study, we examined if LSN862 (LSN), a novel non-thiazoledinedione partial PPAR-**γ** agonist, was neuroprotective in a mouse model of Parkinson's disease (PD) and assessed possible mechanisms of action. LSN (3, 10, or 30 mg/kg) or vehicle was orally administered daily to C57BL/6 and antioxidant response element-human placental alkaline phosphatase (ARE-hPAP) reporter mice 3 days prior to 1-methyl-4-phenyl-1,2,3,6-tetrahydropyridine (MPTP; 30 mg/kg, i.p. ×  5 days) or PBS administration. LSN elicited a dose-dependent preservation of dopaminergic nigrostriatal innervation that was not associated with inhibition of MPTP metabolism or activation of Nrf2-ARE, although changes in NQO1 and SOD2 mRNA were observed. A significant dose-dependent downregulation in MAC-1 and GFAP positive cells was observed in MPTP + LSN-treated mice as well as significant downregulation of mRNA expression levels of these inflammatory markers. MPTP-induced increases in PPAR-**γ** and PGC1**α** expression were ameliorated by LSN dosing. Our results demonstrate that oral administration of LSN is neuroprotective against MPTP-induced neurodegeneration, and this effect is associated with downregulation of neuroinflammation, decreased oxidative stress, and modulation of PPAR-**γ** and PGC1**α** expression. These results suggest that LSN can be a candidate alternative non-thiazoledinedione partial PPAR-**γ** agonist for neuroprotective treatment of PD.

## 1. Introduction

Neuroinflammation plays a key role in nigral dopaminergic (DA) cell loss in Parkinson's disease (PD; [1]). Microglia serve as resident immune cells of the nervous system, and under normal conditions they monitor the environment of the brain in a resting state. However, in response to trauma or insult, microglia become activated, exhibiting phagocytic morphology and upregulation of CD1 and cell adhesion molecules such as MAC-1 (CD11b) and CD54 [1]. When activated for a prolonged period of time, microglia release a cascade of proinflammatory cytokines such as TNF-*α*, IL-1*β*, and IL-6, which may lead to mitochondrial dysfunction and cell death [1]. Activated microglia are present at a high density in the substantia nigra of patients with PD [2, 3]. Nigral DA neurons seem to be particularly susceptible to inflammation due to a number of factors including decreased glutathione levels (reducing antioxidant ability [4], diminished redox activity [5], high density of neuromelanin [6], and elevated iron concentrations [7]). While microglia are the chief mediators of inflammation in the brain, astroglia are part of the inflammatory response as well. Astrogliosis is observed after environmental insult and injury, and it is characterized by upregulation of glial fibrillary acidic protein (GFAP). 1-Methyl-4-phenyl-1,2,3,6-tetrahydropyridine (MPTP)-treated mice like PD patients present reactive microglia and astrogliosis in the nigrostriatal system [8, 9]. 

Peroxisome proliferator activated receptor-gamma (PPAR-*γ*) is one of three PPAR isoforms. PPAR-*γ* modulates gene expression in a ligand dependent and independent manner [10] and has been previously reported to attenuate proinflammatory genes while decreasing gliosis [11, 12]. PPAR-*γ* coactivator-1 alpha (PGC1*α*), which functions as a mitochondrial master sensor of energy metabolism, also seems to have anti-inflammatory and antioxidative properties [13, 14]. Agonists to PPAR-*γ*, such as the thiazolidinediones rosiglitazone (Avandia) and pioglitazone (Actos), are emerging as potential treatments for PD, as they seem to modulate these pathways. Rosiglitazone administration to mice treated with MPTP and the renal clearance inhibitor probenecid induced neuroprotection of TH-ir nigral neurons, inhibited motor and olfactory dysfunctions, and decreased neuroinflammatory markers [15]. Similarly, pioglitazone dosing to MPTP-treated mice induced preservation of DA nigral neurons, decreased activation of microglia, and attenuated nitric oxide-mediated toxicity [16, 17]. In Parkinsonian nonhuman primates, administration of pioglitazone restored motor function, prevented nigrostriatal dopaminergic cell loss, and modulated neuroinflammation [18]. In addition to anti-inflammation, pioglitazone has been reported to elicit neuroprotective effects against MPTP by inhibiting MAO-B [19] and by affecting antioxidative pathways [20]. 

Although rosiglitazone and pioglitazone are promising therapeutic candidates, they may present disadvantages for chronic administration to susceptible populations, such as potentially harmful cardiovascular effects [21–23]. Compared to rosiglitazone, pioglitazone seems to present a lower risk [21, 24], yet it would be desirable to have alternative PPAR-*γ* agonists devoid of those complications. 

LSN862 (LSN; Eli Lilly Ltd.) is a novel non-thiazolidinedione partial PPAR-*γ* agonist. It is easily absorbed after oral dosing and, like other PPAR-*γ* agonists, has a high affinity for PPAR-*γ* and a low, but significant, affinity for PPAR-*α* (see [25] for LSN binding characterization). Interestingly, PPAR-*α* activation has been associated with neuroprotection in PD mice models (e.g., [26]). Here, we report our evaluation of LSN as a neuroprotective compound in a MPTP murine model of PD and its possible mechanisms of action.

## 2. Materials and Methods

### 2.1. Animals and Procedures

Male C57BL/6 mice (8–10 weeks of age, 20–25 g) were obtained from Jackson Laboratories (Bar Harbor, Maine). Antioxidant response element-human placental alkaline phosphatase (ARE-hPAP) reporter mice (8–10 weeks of age, 20–25 g) were created using the NAD(P)H:quinone oxidoreductase (NQO1) promoter upstream of an hPAP reporter construct [27]. Animals were housed at room temperature under a 12 hr light-dark cycle and had access to food and water ad libitum. All efforts were made to minimize the number of animals used and ameliorate their suffering. This study was performed in strict accordance with the recommendations in the Guide for the Care and Use of Laboratory Animals of the National Institutes of Health. All protocols were approved by the Institutional and Animal Care Committee at the University of Wisconsin-Madison.

 Mice were administered MPTP-HCl in saline (or saline alone) in a subchronic paradigm (30 mg/kg i.p. for 5 days). Starting three days prior to MPTP or PBS, dosing animals received daily oral administration of vehicle (Neobee oil; Spectrum chemical) or 3, 10, or 30 mg/kg of LSN. LSN and vehicle dosing were done 8 hrs before MPTP and PBS. To test neuroprotective efficacy, daily treatments for C57BL/6 mice continued for twenty-one days after the last MPTP administration, and, to test ARE activation, ARE-hPAP mice were sacrificed seven days after the last administration. Effects of LSN on MPTP metabolism were evaluated in mice euthanized 15 min or 90 min after the last dose of MPTP (1 × 5 days at 30 mg/kg, i.p.) and eight hours after LSN (30 mg/kg) or placebo treatment. Time-dependent evaluation of mRNA expression was evaluated in mice euthanized 24 hrs, 72 hrs, or 21 days after the last dose of MPTP and 24 hrs after the last treatment of LSN (30 mg/kg) or placebo. At the end of each experiment, mice were killed by CO_2_ inhalation. 

For immunohistochemical experiments, animals were transcardially perfused with ice-cold saline followed by 4% paraformaldehyde (PFA); their brains were extracted, postfixated for 24 hrs in 4% PFA, and then transferred to graded sucrose. Brains were cut with a sliding microtome in 40 *μ*m sections and stored at −20°C in cryoprotectant solution until staining. 

For biochemical and RT-qPCR analysis, animals were perfused transcardially with ice-cold saline; after brain extraction, the ventral midbrain and striatum were manually dissected out and stored at −80°C until processing. 

### 2.2. Pharmacokinetic Analysis of LSN862

To determine systemic and brain exposure to LSN862, C57BL/6 mice were orally administered 5 mg/kg of LSN862. The dosing suspension was prepared in a vehicle consisting of 1% CMC, 0.5% SLS, 0.085% povidone, and 0.05% Dow Corning Antifoam 1510 in purified water. Following oral administration, whole blood and brain samples were collected for the measurement of plasma and brain concentrations from 3 mice per timepoint at 0.25, 0.5, 1, 2, 4, 8, 12, 24, and 48 hours postdose. Plasma was obtained by centrifugation of the whole blood and brain samples were collected following whole body perfusion with approximately 10 mL of saline. Samples were stored at approximately −70°C until being analyzed using a bioanalytical LC/MS/MS method.

### 2.3. Immunohistochemistry

Coronal brain sections were used for immunohistochemical staining as previously described [18, 28]. Briefly, endogenous peroxidase activity was removed with a 20-minute incubation in 0.1 M sodium periodate. After 3 × 10 minute washes in PBS plus 0.05% Triton-X (dilution media), background staining was blocked with a 1-hour incubation in a Tris buffered saline (TBS) solution containing 3% normal serum, 2% bovine serum albumin, and 0.05% Triton X-100. The sections were incubated with a primary antibody for 24 hrs at room temperature, then incubated for 1 hour in biotinylated secondary antibody (1 : 200; Vector Laboratories, Burlingame, CA, USA). After 12 × 10 minute washes in dilution media, the sections were placed in the avidin biotin (ABC, “Elite” kit, Vector Laboratories) substrate for 75 minutes. Sections were then washed in a 0.1 M imidazole/1.0 M acetate buffer, pH 7.4, and then reacted in a chromagen solution containing 0.05% 3,3′-diaminobenzidine and 0.05% H_2_O_2_. Antibodies used include tyrosine hydroxylase (TH 1 : 1,000; Millipore, Billerica, MA, USA), mouse antibody cd11b (MAC-1; 1 : 1,000; AbD Serotec, Oxford, UK), and glial fibrillary acid protein (GFAP; 1 : 1000; DakoCytomation, Glostrup, Denmark). Adjacent sections were also stained with Nissl for general morphological evaluation and further quantification of nigral neurons.

### 2.4. Optical Density Quantification

The optical density (OD) of TH immunoreactive (ir) fibers was quantified in four equally spaced coronal sections within ventral, medial, and dorsal sections of the striatum using NIH ImageJ software. Brain images were captured in a Nikon Super Coolscan 4000 ED Film & Slide Scanner. ImageJ was calibrated using a step tablet, grey scale values were converted to OD units using the Rodbard function, and the mean OD for each area of interest was recorded.

Immunoreactivity of MAC-1 and GFAP was quantified in four equally spaced coronal sections at the level of the substantia nigra. Brain images were captured using a Nikon E800 microscope equipped with a SPOT camera and evaluated using NIH Image J software. The images were converted to an 8-bit grayscale and calibrated using a step tablet. The area above the threshold was determined using ImageJ's Triangle function, background subtracted, and the area above the threshold was recorded. 

### 2.5. Stereological Cell Counts

The total number of TH-ir and Nissl stained neurons in the right and left substantia nigrae was calculated using unbiased stereological cell-counting methods [29–32]. Four equally spaced sections from each subject containing the substantia nigra were used for analysis. The optical dissector system consisted of a computer assisted image analysis system including a Zeiss Axioplan 2 imaging photomicroscope (Carl Zeiss, Inc.) hard-coupled to a MAC5000 high precision computer-controlled *x*-*y*-*z* motorized stage and a MicroFire CX9000 camera (Optronics, Goleta, CA, USA). Neuronal counts were performed using Stereo Investigator Version 7.5 (MicroBrightField, Williston, VT, USA). The substantia nigra was outlined under low magnification (2.5x). The total number of TH-ir and Nissl positive nigral neurons within the counting frame was counted using a 100x oil immersion objective. The substantia nigra limits were defined by the caudal edge of the mammillary bodies rostrally, the cerebral peduncle ventrally and laterally, and the third cranial nerve rootlets medially. The grid size used was *x* = 200 *μ*m, and *y* = 200 *μ*m and the counting frame size was 60 *μ*m × 60 *μ*m. Neurons were only counted within a 20 *μ*m height of tissue, with guard heights of 2 *μ*m at the top and remaining *μ*m at the bottom of each section.

### 2.6. HPLC Analysis

Striatal levels of dopamine (DA) and DA metabolites, DOPAC, and HVA were measured using high performance liquid chromatography electrochemical detection (HPLC/ECD). Samples were weighed, homogenized in ice-cold perchloric acid (0.3 M), and centrifuged, and a 200 *μ*L aliquot of sample was analyzed using a coulometric electrochemical detector (Choulochem III; ESA, Chelmsford, MA, USA). The guard cell was set to 250 mV. Electrodes 1 and 2 were adjusted to −250 mV and +270 mV, respectively. Chromatographic separations were performed on a column, 250 mm × 4.6 mm, run at room temperature (Beckman, Indianapolis, IN, USA). The mobile phase consisted of 75 mM sodium dihydrogen phosphate, 1.7 mM 1-octanesulfonic acid, 100 *μ*L/L of triethylamine, 25 *μ*M of EDTA, and 10% acetonitrile, and was adjusted to 3.0 with phosphoric acid. These solutions were prepared in HPLC grade water, filtered through a 0.22 *μ*m membrane under vacuum, and pumped at a rate of 0.6 mL/min, producing a background pressure of 68 bars. Two-hundred microliter aliquots were injected by an autoinjector with cooling module set at 4°C. The levels of DA, DOPAC, and HVA in experimental samples were determined using standard curves created by injection of known concentrations of each substance. 

### 2.7. Western Blot Analysis

Striatal TH protein levels were quantified by Western blot analysis [33]. Striatal protein extracts from ARE-hPAP mice were homogenized in ice cold lyses buffer (50 mM TRIS, pH 7.4; 150 mM NaCl; 1 mM EDTA) and supplemented with a Complete Mini, EDTA-free protease inhibitor cocktail tablet (Roche Applied Science, Basal, Swizerland). Supernatants were collected upon centrifugation and stored at −80°C until analysis. Protein concentrations were determined using the BCA assay kit (Pierce, Rockford, IL, USA). Twenty to 30 *μ*g of protein per lane was loaded onto a 10% sodium dodecyl sulfate-polyacrylamide electrophoresis gel and transferred onto a PVDF membrane (Millipore, Billerica, MA, USA). PVDF membranes were blocked with 5% BSA in Tris-buffered saline Tween 20 (TBST) at room temperature for 1 hr and then incubated with primary antibody (rabbit polyclonal anti-TH; Chemicon, Billerica, MA; 1 : 2,000, mouse monoclonal anti-beta-actin (1 : 5,000, Abcam, Cambridge, MA, USA) overnight at 4°C. The PVDF membrane was then washed 3× in TBST followed by 1 hr incubation at room temperature in TBST in a 1 : 10,000 dilution of horseradish peroxidase labeled anti-rabbit or anti-mouse (Amersham, Piscataway, NJ, USA). After washing in TBST, bands were visualized using enhanced chemiluminescence (Super Signal West Pico; Pierce, Rockford, IL, USA) and the signal exposed with Hyperfilm (Amersham, Piscataway, NJ, USA). Gel bands were quantified using ImageJ (NIH) and normalized to beta-actin.

### 2.8. MAO-B Activity and MPP^+^ Detection Assays

Potential modulation of MPTP metabolism by LSN was evaluated in striatal tissue by quantification of MAO-B activity and MPP^+^ levels [34]. For MAO-B activity, tissue was processed using an Amplex Red monoamine oxidase enzyme immunoassay kit (Invitrogen, Carlsbad, CA, USA) according to the manufacturer's instructions on a 96-well plate and measured with a fluorescence plate reader (Molecular Devices, Spectra Max M2, Sunnyvale, CA, USA). For the MPP^+^ assay, striata were homogenized in 9 volumes of 5% trichloroacetic acid. Fifty *μ*L of homogenized tissue was injected into the HPLC-UV detector (model no. 168 Beckman) with a reverse phase C18 column (100 × 4.6 mm) using a mobile phase consisting of 9 volumes 50 mM KH_2_PO_4_ and 1 volume acetonitrile, pH adjusted to 3.2. The flow rate was set at 1.8 mL/min using a solvent delivery pump. All instruments were connected to a computer, and the samples were run and data-analyzed by detection software (Beckman, Nouveau, System Gold, Indianapolis, IN, USA).

### 2.9. hPAP Activity Assay

Activation of the ARE-Nrf2 pathway was measured in ARE-hPAP reporter mice by quantification of alkaline phosphatase activity as previously described [27]. Briefly, striatum and substantia nigra protein extracts were lysed as described for Western blotting. 10 *μ*L of the lysed tissue was added to 40 *μ*L of TMN (50 mM Tris, 5 mM MgCl_2_, and 100 mM NaCl) with 4% CHAPS. To inactivate endogenous phosphatase activity, samples were heated to 65°C in 0.2 M diethanolamine (DEA) buffer for 20–30 min. Following heat inactivation, CSPD and Emerald (Applied Biosystems, Carlsbad, CA, USA) were used as luminescent substrate and enhancer, respectively. A Berthold Orion microplate luminometer was used to measure samples in a 96-well plate. Values were corrected to those of ARE-hPAP negative mice and normalized by protein concentration.

### 2.10. Real-Time PCR

Striatal and nigral mRNA expression of dopaminergic, inflammatory, antioxidative, and other genes of interest (see Supplementary Table 1 for primers listed in detail in supplementary material available online at http://dx.doi.org/10.1155/2013/582809) were evaluated using real-time quantitative PCR (RT-qPCR). Total RNA was extracted from with TRIZOL according to manufacturer's instructions (Invitrogen Corporation) and treated with RNeasy MinElute Cleanup (Qiagen, Venlo, Netherlands). RNA quality and concentration were measured using the RNA 6000 Nano chip and analyzed with the Agilent 2100 Bioanalyzer (Agilent Technologies, Foster City, USA) or quantified with a Nanodrop ND-1000 Spectrophotometer (Thermo Scientific, Wilmington, NC, USA) and quality evaluated with the Bio-Rad Experion Automated Electrophoresis System (Bio-Rad, Hercules, CA, USA). One *μ*g of RNA (RIN or RQI > 7.7) was reverse-transcribed into cDNA using the reverse transcription system (Promega, Madison, WI) according to manufacturer's instructions. Quantitative PCR of the cDNA was performed in a Light Cycler 480 Real Time PCR (Roche Applied Science, Basel, Switzerland) or StepOnePlus Real Time PCR System (Applied Biosystems, Carlsbad, California) with SYBR Green Master Mix according to manufacturer's instructions. All PCR product quantification was subject to relative standard curves. Efficiencies of amplification were limited to between 1.8 and 2.2 with an error of <0.2. To ensure accuracy and precise calibration, beta-actin was used as an internal control. No significant differences were found in beta-actin with respect to treatment group. 

### 2.11. Statistical Analysis

 All data was collected and analyzed by investigators blind to the treatment groups. For each experiment, animals were randomly assigned to each treatment group. Analysis of variance followed by LSD post hoc pairwise comparisons or independent *t*-tests was used to assess differences between groups. A two-way ANOVA with repeated measures was used to evaluate RT-qPCR data between groups over time followed by LSD post hoc pairwise comparisons. A *P* value of <0.05 was considered statistically significant for all analyses. Data are presented as mean ± SEM. All statistics were performed using SPSS version 18.0 software (IBM, Sommers, NY, USA). 

## 3. Results

### 3.1. LSN862 Readily Crossed the Blood Brain Barrier

Following a 5 mg/kg dose of LSN862 in mice, peak plasma and brain concentrations of LSN862 occurred 1 hour after the dose. Maximum observed plasma and brain concentrations (*C*_max⁡_) were 13223 and 382 ng/mL, respectively. These data indicate systemic exposure of LSN862 and that the compound crosses the blood brain barrier.

### 3.2. LSN862 Preserved Dopaminergic Nigrostriatal Innervation

**Figure 1:**
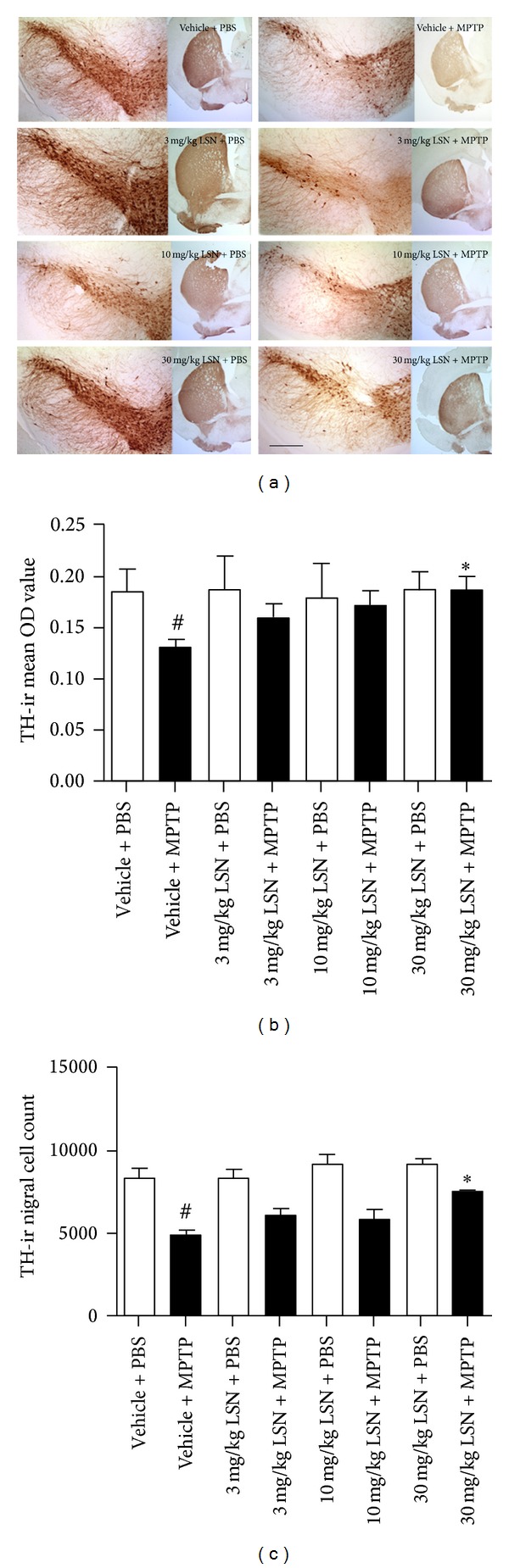
LSN862 (LSN) conferred neuroprotection against MPTP nigrostriatal loss in a dose-dependent manner. Mice brains were evaluated 21 days after completing MPTP or PBS challenge and 24 hrs after daily treatment with LSN doses or vehicle. Quantifications were performed in the following groups: vehicle + PBS (*n* = 7); vehicle + MPTP (*n* = 7); 3 mg/kg LSN + PBS (*n* = 4); 3 mg/kg LSN + MPTP (*n* = 4); 10 mg/kg LSN + PBS (*n* = 4); 10 mg/kg LSN + MPTP (*n* = 6); 30 mg/kg LSN + PBS (*n* = 6); and 30 mg/kg LSN + MPTP (*n* = 7). (a) Coronal images at the level of striatum (ST) and substantia nigra (SN) immunostained for tyrosine hydroxylase (TH). Scale bar: ST = 1 mm, SN = 200 *μ*m. (b) TH optical density (OD) of striatal fibers (mean ± SEM). Independent *t*-test ^#^ *P* < 0.05 (vehicle + PBS versus vehicle + MPTP), *P < 0.05 (vehicle + MPTP versus 30 mg/kg LSN + MPTP). (c) Stereological cell quantification of TH positive nigral neurons (mean ± SEM; *n* = 4–7 per group) ANOVA (Fisher's LSD post hoc) ^#^ *P* < 0.01 (vehicle + PBS versus vehicle + MPTP), *P < 0.01 (vehicle + MPTP versus 30 mg/kg LSN + MPTP).

**Figure 2:**
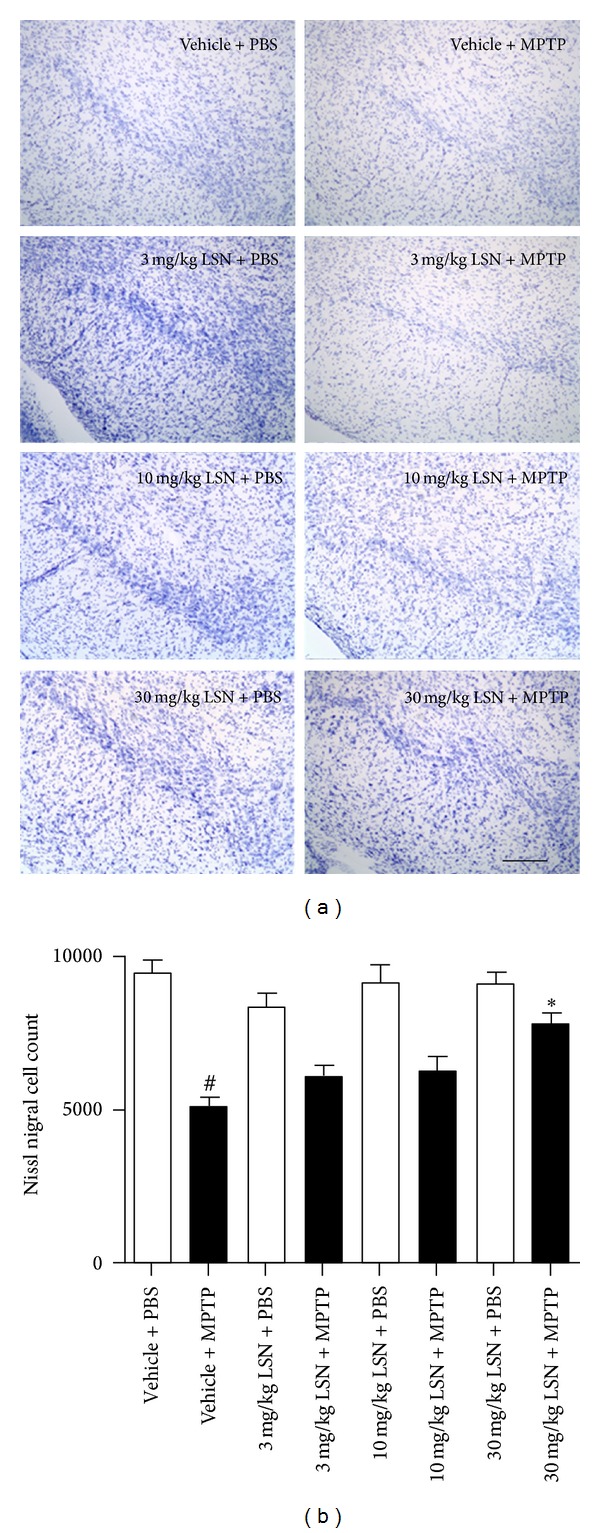
LSN862 (LSN) induced neuroprotection against MPTP not upregulation of the dopaminergic phenotype. Mice were treated with MPTP or PBS and LSN doses or vehicle and euthanized 21 days later. (a) Coronal images at the level of the substantia nigra (SN) stained for Nissl. Scale bar = 100 *μ*m. (b) Stereological cell quantification of Nissl positive SN cells (mean ± SEM; *n* = 4–7 per group). ANOVA (Fisher's LSD post hoc) ^#^ *P* < 0.001 (vehicle + PBS versus vehicle + MPTP), *P < 0.001 (vehicle + MPTP versus 30 mg/kg LSN + MPTP).

Qualitative evaluation of TH immunohistochemistry 21 days post-MPTP showed a loss of dopaminergic positive fibers and nigral cells in MPTP-treated mice compared to PBS controls which seemed to be ameliorated by LSN (Figure 1(a)). Optical density (OD) quantification of TH immunoreactive (ir) striatal fibers confirmed a significant reduction in MPTP-treated mice (Figure 1(b)) with a significant preservation in animals treated with 30 mg/kg of LSN. Stereological TH-ir nigral cell counts of mice treated with vehicle + MPTP or LSN at 3, 10, and 30 mg/kg + MPTP showed a significant reduction in TH-ir neurons compared to vehicle + PBS. Comparison between 30 mg/kg LSN + MPTP versus vehicle + MPTP-treated animals demonstrated preservation of TH-ir nigral neurons in the LSN group (Figure 1(c)). The findings of significant nigral cell loss by MPTP and preservation by 30 mg/kg of LSN were confirmed by quantification of Nissl-stained nigral sections (Figure 2).

**Figure 3:**
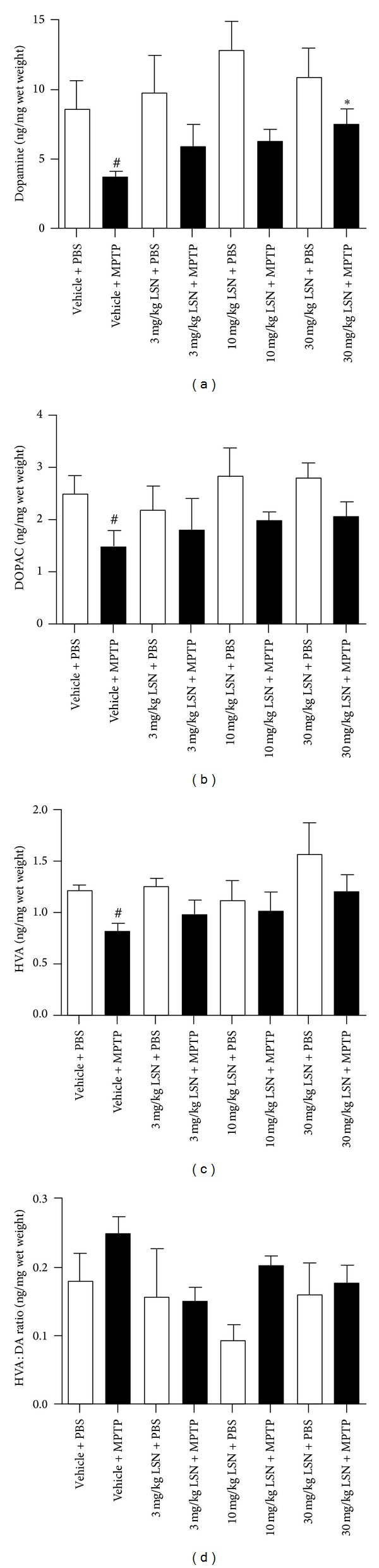
LSN862 (LSN) induced protection of striatal catecholamines against MPTP dopaminergic loss. HPLC analysis of striatal catecholamine levels was performed in mice tissues obtained 21 days after treatment with MPTP or PBS and LSN or vehicle (mean ± SEM). (mean ± SEM; *n* = 4–7 per group). ANOVA (Fisher's LSD post hoc). (a) Dopamine: ^#^ *P* < 0.05 (vehicle + PBS versus vehicle + MPTP), *P < 0.05 (vehicle + MPTP versus 30 mg/kg LSN + MPTP). (b) DOPAC: ^#^ *P* < 0.05 (vehicle + PBS versus vehicle + MPTP). (c) HVA: ^#^ *P* < 0.05 (vehicle + PBS versus vehicle + MPTP). (d) DOPAC/HVA ratio.

HPLC analysis of catecholamine levels in striatal tissue demonstrated MPTP-induced depletion of DA in vehicle + MPTP-treated mice compared to PBS controls (Figure 3(a)), which was protected by treatment with 30 mg/kg of LSN. MPTP significantly decreased striatal DOPAC (Figure 3(b)) and HVA (Figure 3(c)) levels in vehicle + MPTP-treated mice compared to vehicle + PBS controls, but these metabolites were not significantly affected by LSN. 

### 3.3. LSN862 Did Not Affect MPTP Metabolism

After we identified 30 mg/kg of LSN as neuroprotective, we used this dose to assess if LSN effects were due to inhibition of MPTP metabolism in the subchronic paradigm. HPLC analysis of MPP^+^ levels in striatal tissue obtained 15 or 90 minutes after the last (5th) injection of MPTP and 8 hours after the last (8th) oral of LSN did not show significant differences between vehicle and LSN-treated groups (Table 1). Similarly, the MAO-B immunoassay did not reveal significant differences in striatal MAO-B activity after LSN treatment compared to vehicle. 

### 3.4. LSN862 Affected MPTP-Induced Striatal and Nigral NQO1 and Nigral SOD2 mRNA

To determine if LSN effects were related to modulation of oxidative stress response, we first assess the Nrf2-ARE pathway in ARE-hPAP reporter mice treated with MPTP or PBS and vehicle or 30 mg/kg of LSN. Western blot of striatal TH protein levels (Supplementary Figure 1(a)) showed a decrease in TH in MPTP samples compared to PBS that was ameliorated by LSN dosing, which was confirmed by densitometric analysis of TH relative to *β*-actin (Supplementary Figure 1(b)). Striatal catecholaminergic evaluation by HPLC also showed a significant loss of striatal catecholamines in ARE-hPAP MPTP-treated mice compared to PBS controls, which was prevented with LSN dosing (Supplementary Figures 1(c)–(1e)). Evaluation of hPAP levels in these mice revealed a MPTP-induced significant decrease in striatal (Supplementary Figure 2(a)) and an increase in nigral (Supplementary Figure 2(b)) hPAP, but these changes were not affected by LSN treatment. 

To assess if the neuroprotective effects of LSN were mediated by an antioxidant response at the transcription level, we evaluated striatal and nigral mRNAs expression of antioxidant markers (Supplementary Tables 2 and 3). NAD(P)H:quinone oxidoreductase 1 (NQO1) striatal and nigral mRNA expression was significantly upregulated 24 hrs post-MPTP compared to saline controls, and only striatal levels were attenuated by 30 mg/kg of LSN. No significant differences were observed at 72 hrs or 21 days. MPTP induced significant increases in other endogenous genes regulated by the Nrf2-ARE pathway, including glutamate cysteine-ligase, catalytic subunit (GCLC), glutamate cysteine-ligase, and modifier subunit (GCLM); however, LSN treatment did not affect them (Supplementary Tables 2 and 3).

**Figure 4:**
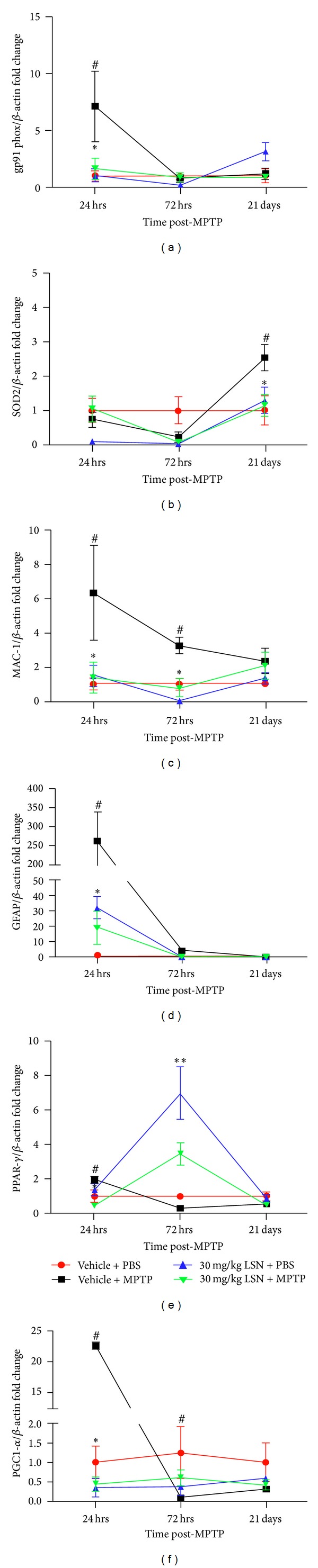
LSN862 (LSN) affected mRNA expression at 24 hrs, 72 hrs, and 21 days post-MPTP. Quantitative PCR analysis of nigral (a) gp91 phox, (b) SOD2, (c) MAC-1, and (d) GFAP and striatal (e) PPAR-*γ*, (f) PGC1*α* mRNA levels. The groups were vehicle + PBS (24 hrs: *n* = 5; 72 hrs: *n* = 4; 21 days: *n* = 4); vehicle + MPTP (24 hrs: *n* = 5; 72 hrs: *n* = 5; 21 days: *n* = 6); 30 mg/kg LSN + PBS (24 hrs: *n* = 4; 72 hrs: *n* = 3; 21 days *n* = 4; 30 mg/kg LSN + MPTP (24 hrs: *n* = 5; 72 hrs *n* = 4; 21 days *n* = 5). Data is expressed as fold change from vehicle + PBS controls relative to *β*-actin (mean ± SEM). Two-way ANOVA with repeated measures, ^#^ *P* < 0.05 (vehicle + MPTP versus vehicle + PBS), *P < 0.05 (vehicle + MPTP versus 30 mg/kg LSN + MPTP), and **P < 0.05 (vehicle + PBS versus 30 mg/kg LSN + PBS).

 Nigral but not striatal superoxide dismutase-2 (SOD2) mRNA levels were detected at 24 and 72 hrs, peaking at 21 days post-MPTP when significant differences between groups were found (Figure 4(b), Supplementary Tables 2 and 3). At that time-point, MPTP-treated mice presented a significant increase in mRNA expression compared to saline controls, which LSN attenuated. 

### 3.5. LSN862 Attenuated MPTP-Induced Neuroinflammation

**Figure 5:**
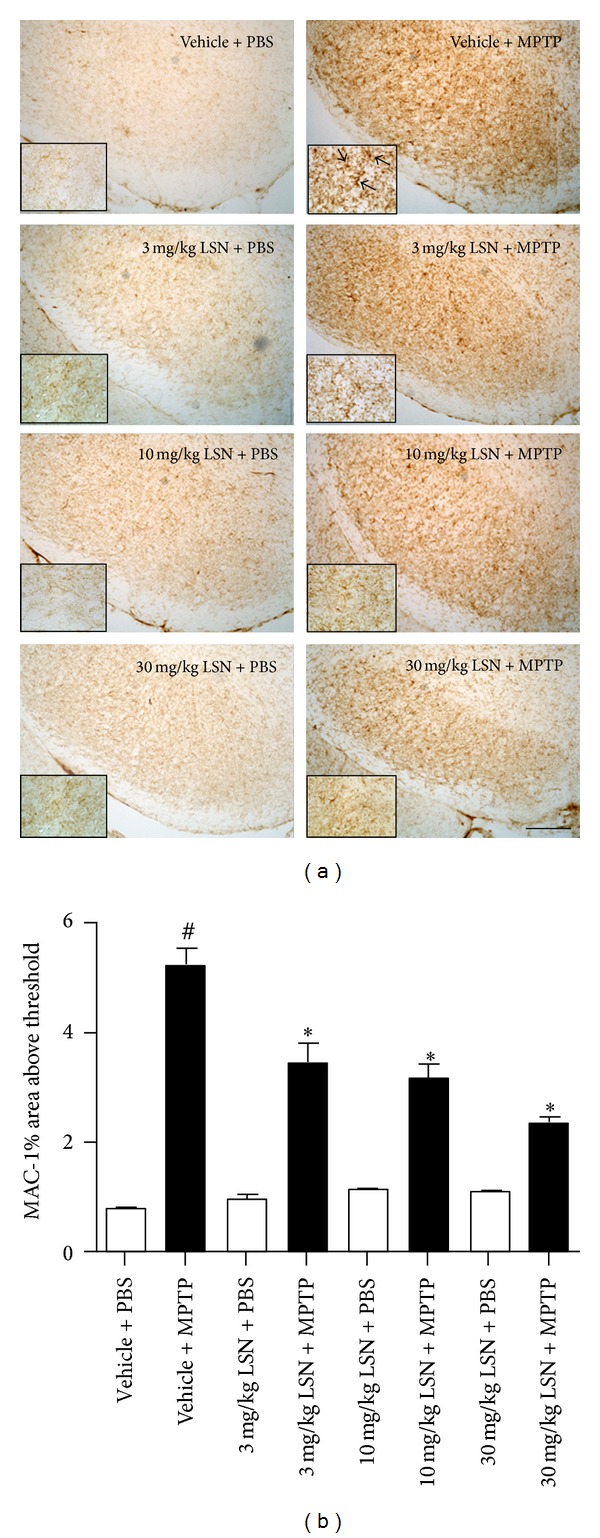
LSN862 (LSN) prevented MPTP-induced microglial activation. Mice brains were evaluated 21 days after completing MPTP or PBS challenge and 24 hrs after daily treatment with LSN. (mean ± SEM; *n* = 4–7 per group). (a) Coronal images at the level of the substantia nigra (SN) immunostained for MAC-1 (cd11b). Insets correspond to high magnification images. Scale bar: low magnification = 100 *μ*m, high magnification = 25 *μ*m. (b) Quantification of nigral MAC-1 immunoreactivity. ANOVA Fisher's LSD post hoc, ^#^ *P* < 0.05 (vehicle + PBS versus vehicle + MPTP), *P < 0.05 (vehicle + MPTP versus 30 mg/kg LSN + MPTP).

**Figure 6:**
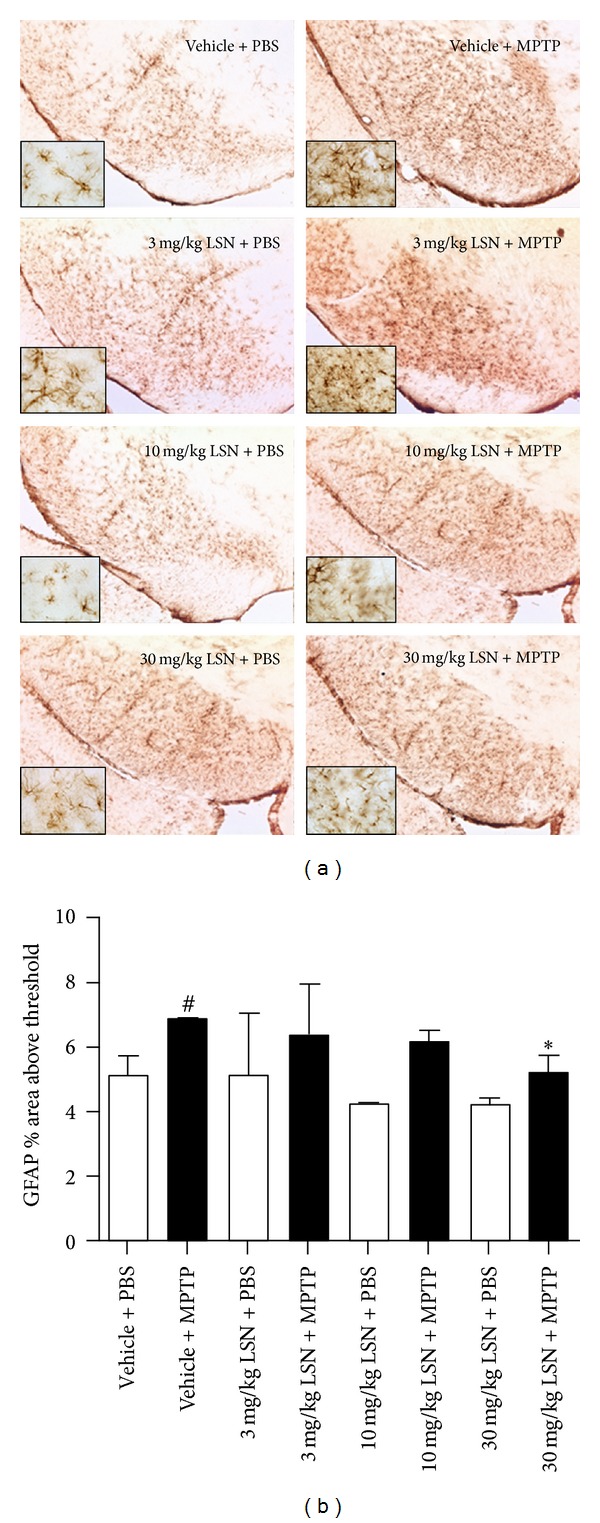
LSN862 (LSN) inhibited MPTP-mediated astrogliosis. Mice brains were evaluated 21 days after completing MPTP challenge and 24 hrs after daily treatment with LSN. (a) Images at the level of the substantia nigra (SN) immunostained for GFAP. Insets correspond to high magnification images. Scale bar: low magnification = 100 *μ*m, high magnification = 25 *μ*m. (b) Quantification of nigral GFAP immunoreactivity (means ± SEM; *n* = 4–7 per group); ANOVA (Fisher's LSD post hoc), ^#^ *P* < 0.05 (vehicle + PBS versus vehicle + MPTP), *P < 0.05 (vehicle + MPTP versus 30 mg/kg LSN + MPTP).

Immunostaining of inflammatory markers in the substantia nigra 21 days post-MPTP showed MAC-1-ir typical of reactive microglia (Figure 5(a)) as well as GFAP-ir astrogliosis (Figure 6(a)). Multiple comparisons analysis of OD quantification revealed that treatment with LSN elicited a dose-dependent significant decrease of nigral MAC-1-ir in animals treated with 3, 10, or 30 mg/kg + MPTP compared to vehicle + MPTP-treated animals (Figure 5(b)). Nigral GFAP-ir was also significantly decreased by 30 mg/kg LSN compared to vehicle + MPTP (Figure 6(b)).

Striatal and nigral MAC-1 and GFAP mRNA expression was evident by 24 hrs in MPTP-treated mice compared to saline controls and was significantly reduced by LSN. At 72 hrs, MAC-1 nigral levels (but not striatal) were also significantly decreased by LSN (Figure 4(c)), while GFAP striatal and nigral (Figure 4(d)) levels were both significantly decreased by LSN. By 21 days post-MPTP, striatal and nigral MAC-1 and GFAP mRNA levels were reduced to near basal levels, and no effect of treatment was observed (Supplementary Tables 2 and 3). 

 mRNA expression of the NADPH proinflammatory enzymatic subunits gp91phox and p67phox were also affected by LSN although following different patterns (Supplementary Tables 2 and 3). A significant change in gp91phox striatal and nigral (Figure 4(a)) levels were only found 24 hrs post-MPTP, when they peaked compared to PBS controls, and LSN prevented the upregulation. Striatal levels of p67phox mRNA was significantly elevated 21 days post-MPTP, which was attenuated by LSN treatment (Supplementary Table 2). Within the nigra, the MPTP-induced increase was significantly attenuated by LSN treatment at 24 and 72 hrs (Supplementary Table 3).

### 3.6. Effect on PPAR-*γ* on Select Protein Kinases by LSN862 Dosing

 Protein kinases AMPK and MAPK are known to induce activation of PGC1*α* and, consequently, PPAR-*γ*. Striatal AMPK mRNA presented differences between treatment groups over time (Supplementary Table 2). At 24 hrs MPTP-injected mice had a robust increase in expression that was attenuated by LSN. Interestingly, striatal AMPK expression was also increased at 24 hrs in PBS-injected mice administered LSN. While no differences in AMPK expression were found at 72 hrs between vehicle-treated mice with MPTP or PBS, LSN increased striatal AMPK expression in MPTP-injected mice. By 21 days, there was no difference between treatment groups. Striatal MAPK mRNA expression was significantly increased at 24 hrs in saline-injected mice administered LSN versus vehicle-treated controls. By 72 hrs, levels were equivalent to vehicle + PBS controls. In the substantia nigra, AMPK and MAPK mRNA expression showed no significant changes overtime or by treatment (Supplementary Table 3). 

### 3.7. PPAR-*γ* and PGC1*α* MPTP-Induced Changes Are Affected by LSN862 Dosing

PPAR-*γ* (Figure 4(e)) and PGC1*α* (Figure 4(f)) mRNA changes were only observed in the striatum, not in the substantia nigra (Supplementary Tables 2 and 3). At 24 hrs, PPAR-*γ* mRNA was significantly upregulated in MPTP-treated mice versus saline controls and decreased by LSN. At 72 hrs, levels diminished in MPTP-injected mice and were similar to PBS controls although a significant increase was found in PBS-injected mice administered LSN versus vehicle + PBS controls. Striatal PPAR-*γ* mRNA was elevated in MPTP mice treated with LSN but was not significantly different compared to MPTP mice treated with PBS. At 21 days post-MPTP, no differences between treatment groups were observed in striatal expression. PGC1*α* mRNA levels peaked at 24 hrs in MPTP-treated mice versus saline controls, which LSN attenuated. At 72 hrs, there was a trend toward a decrease in PGC1*α* levels in MPTP-treated animals versus vehicle + PBS-treated controls that did not reach statistical significance. By 21 days post-MPTP, PGC1*α* expression diminished, and no differences were observed between treatment groups.

## 4. Discussion

Our results document three main new findings. First, after oral administration the novel non-thiazolidinedione partial PPAR-*γ* agonist LSN induced dose-dependent neuroprotection against MPTP nigrostriatal degeneration in mice. Second, LSN-induced neuroprotection was associated with early downregulation of glial reactivity and oxidative stress response. Third, increases in PPAR-*γ* and PGC1*α* mRNA expression after MPTP were ameliorated by LSN treatment. 

The preservation of nigral TH-ir and Nissl positive cell counts confirms that treatment with LSN enhanced the survival of DA nigral neurons, instead of inducing upregulation of the dopaminergic phenotype [35]. We chose a subchronic dosing paradigm of MPTP as it induces a well-characterized model of PD, in which nigral cell death occurs in a protracted manner with predominantly apoptotic mechanisms compared to the necrotic neuronal death observed with acute dosing [35–38]. The paradigm of three days of pretreatment dosing of a potentially therapeutic drug, although it has limited clinical relevance, is an accepted dosing regimen for initial evaluation of novel neuroprotective strategies in rodents (e.g., [39, 40]). While it could be argued that LSN delayed, rather than protected against cell death, it is unlikely this is the case here as the animals in our study were sacrificed three weeks after the last injection of MPTP, and MPTP-treated controls demonstrated nigral, dopaminergic cell loss. Follow-up studies that start LSN dosing after MPTP challenge may further confirm LSN's neuroprotective properties.

LSN does not seem to induce neuroprotection by affecting MPTP metabolism. MPTP toxicity depends on its transformation by intracerebral MAO-B into its metabolite MPP^+^ [41]. We found that both MAO-B activity and MPP^+^ levels remained unchanged by LSN dosing. The possibility exists that our sampling was not able to capture an LSN effect. We collected brain samples at 90 minutes post-MPTP because it has been documented that at that time point MPP^+^ production reaches its peak [34]; the sampling at 15 minutes was aimed to assess an earlier time point as this was a subchronic MPTP paradigm with daily LSN dosing that should induced a chronic MAO-B inhibition. It could also be argued that LSN induced astroglial dysfunction, which decreased MPTP metabolism (as these cells are the main source of MAO-B) therefore decreasing MPTP toxicity. Yet, this seems unlikely as histological evaluation of GFAP-ir as well as GFAP mRNA levels of PBS + LSN-treated mice compared to PBS + vehicle controls did not show significant differences. While Quinn and colleagues [19] reported that the PPAR-*γ* agonist pioglitazone modulated levels of MPP^+^ and MAO-B, Schintu et al. [15] found no evidence that the related compound rosiglitazone elicited such effects. These reports, in addition to our current data, suggest that modulation of MPTP metabolism by PPAR-*γ* agonists is a compound-specific property. 

 The transient and early response of NQO1 mRNA striatal and nigral expression to MPTP and LSN suggests partial recruitment of the Nrf2 pathway, although not by direct ARE activation, as our tests in ARE-hPAP reporter mice did not show LSN-induced significant changes in hPAP. This differs from reports of rosiglitazone, pioglitazone, and the endogenous PPAR-*γ* agonist 15d-PGJ(2), inducing direct ARE activation in hepatic and hepatoma cell cultures and hyperoxic lung tissue [20, 42, 43] and suggests that LSN has less neuroprotective potential via Nrf2 pathway compared to those agonists. The other antioxidative stress marker noted to change was the mitochondrial antioxidant SOD2. Its nigral mRNA expression levels peaked at 21 days post-MPTP and were inhibited by LSN, suggesting that oxidative stress may have been reduced early after neurotoxin challenge by LSN, therefore inducing a diminished SOD2 response overtime. Our conservative use of animals (which is demonstrated in the small *n* per group) may have limited the detection of changes due to the individual variability in response to MPTP. Further studies with higher numbers as well as experiments focusing on direct oxidative stress measures will be useful to assert the scope of LSN impact on ROS production. 

The early effects of LSN on striatal and nigral mRNA expression of the NADPH-oxidase subunits gp91phox and p67phox suggest an association between decreased oxidative stress and neuroinflammation. Both subunits are increased during oxidative stress and are indirect measures of nitric oxide synthase (iNOS) production, an inflammatory enzyme associated with microglial driven ROS and NO productions [8]. Furthermore, nigral gp91phox mRNA has been shown to be upregulated in response to MPTP, and inhibition of the NADPH-oxidase complex is associated with a reduction in inflammation [9]. 

LSN's effects in brain inflammatory response were detected as early as 24 hrs after the last injection of MPTP. The association between neuroprotection and decreased inflammation observed with LSN dosing is consistent with reports of other PPAR-*γ* as well as PPAR-*α* agonists. Rosiglitazone administration to MPTP-treated probenecid mice induced anatomical and functional neuroprotection while decreasing reactive CD11b microglia [15, 44]. Pioglitazone dosing to MPTP-treated mice was also neuroprotective and reduced CD11b microglia, as well as nitrotyrosine [16, 17], and, in a PD monkey model, neuroprotection was associated with decreased CD68-ir microglia [18]. The anti-inflammatory activity of PPAR-*γ* agonists has been further confirmed in a rodent model of neuroinflammation induced by intracerebral injections of lipopolysaccharide, in which pioglitazone dosing induced a significant reduction in nigral microglial cells [45]. The PPAR-*α* agonist palmitoylethanolamide (PEA) has also shown functional and neuroanatomical protection against MPTP that was associated with decreased iNOS nitrotyrosine [26], which suggests that in our paradigm LSN activation of PPAR-*α* may have contributed to the neuroprotective effect. Although in our study MAC-1 nigral mRNA expression showed no difference between treatment groups at 21 days post-MPTP, we detected a decrease in MAC-1 protein immunoreactivity. It is possible that this discrepancy may be related to posttranslational modification [46, 47]. The findings of a dose-dependent reduction in MAC-1 nigral reactivity and mRNA expression combined with the reduction in the NADPH subunit gp91phox mRNA levels suggest that LSN may inhibit iNOS, consistent with previous reports of PPAR-*γ* and PPAR-*α* modulation of iNOS [16, 26, 48]. 

Markers of micro- and astrogliosis were both present 24 hrs after MPTP. This differs from a previous report in an acute MPTP mouse model [8] that described increased nigral microglial recruitment preceding astroglia. Most likely, our subchronic paradigm hid the differential time-dependent effect. The subchronic paradigm of MPTP intoxication may have also affected the response to LSN treatment. While we found that LSN induced early downregulation of astrogliosis, previous studies with pioglitazone dosing in an acute MPTP model found effects limited to the nigra [17]. However, in a subchronic paradigm [16], a significant reduction in striatal and nigral GFAP positive cells in pioglitazone + MPTP-treated mice was reported. In addition, in a chronic dosing paradigm + probenecid, rosiglitazone attenuated nigral, but not striatal, GFAP-ir cells [15]. It should be noted that in other models of neurodegeneration such as ALS, pioglitazone attenuated astroglial response [49] similar to our findings with LSN. Interestingly, neuroprotection by PPAR-*α* agonist PEA was also associated with decreased nigral GFAP protein [26] 8 days post-MPTP. This suggests that LSN effect on astrocytes maybe related to combined PPAR-*γ* and -*α* activity. It could be argued that LSN generated astroglial dysfunction; however, it is unlikely as no differences were found in striatal and nigral mRNA or GFAP-ir cells between LSN + PBS-treated mice and vehicle + PBS-treated controls.

 Striatal, but not nigral, PPAR-*γ* and PGC1*α* mRNA expression was significantly affected by MPTP and LSN treatments. CNS mapping of PPAR-*γ* expression in rodents has confirmed its presence in the striatum and substantia nigra [50, 51]. Yet, semiquantitative analysis described a more robust presence in the striatum compared to the mesencephalon, suggesting that changes are maybe more detectable in the areas with more abundant expression. In addition, PPAR-*α* presence has only been confirmed in rodent substantia nigra [50, 51] suggesting that maybe the activation of this pathway modulated the effect. It should be noted that, similar to PPAR-*γ* and PGC1*α*, AMPK and MAPK mRNA expression changes were only observed in the striatum, which can be explained as these kinases are linked to activation of the latter and energy metabolism [52–56]. 

The finding that striatal PPAR-*γ* and PGC1*α* mRNA expression increased early after MPTP and LSN attenuated this mRNA expression by 21 days post-MPTP brings forward questions about PGC1*α* levels for successful neuroprotection. In PD brains as well as in the brains of Parkin conditional knockout mice [57] Parkin interacting substrate (PARIS; ZNF746) has been shown to repress PGC1*α* and facilitate neurodegeneration, suggesting that PGC1*α* is needed for survival of dopaminergic neurons. PGC1*α* is a stimulator of mitochondrial biogenesis and respiration, and an increase in ROS triggers PGC1*α* induction. MPTP inhibits mitochondrial complex I increasing ROS, which in turn increases PGC1*α* levels and, seemingly, PPAR-*γ*. In an acute MPTP model, PPAR-*γ* mRNA expression peaked at 7 days post-MPTP, and subsequently decreased to near basal levels [58]. In a chronic model with MPTP and probenecid, PPAR-*γ* was upregulated 3 days post-MPTP [44]. Furthermore, PGC1*α* null mice were more susceptible to MPTP-induced DA cell death compared to wildtype mice [59]. Yet, sustained overexpression of PGC1*α* in rat substantia nigra by gene transfer impaired dopaminergic nigrostriatal function, and, when overexpressed at high levels, PGC1*α* induced neurodegeneration [60]. In our study LSN did not abolish PGC1*α* in response to MPTP but prevented a high peak and facilitated its normalization overtime, probably by stimulating key ROS-detoxifying enzymes like SOD2. These data suggest that nigral cell survival depends on physiological levels of PGC1*α*, rather than high ones, and that LSN partial effects on PPAR-*γ* and its coactivator (in addition to the anti-inflammatory and antioxidative stress properties) may have contributed to nigral neuroprotection. 

 Although we observed changes in the upregulation of PPAR-*γ* and PGC1*α* in parallel to glial activation in LSN + MPTP treated mice, we cannot unequivocally state whether the effects of LSN were PPAR-*γ* dependent or independent [61] and what role the partial PPAR-*γ* activation had in this modulation as compared to rosiglitazone; LSN has 66% recruitment efficacy of PGC1*α* [25]. Several studies have shown that anti-inflammatory effects such as inhibition of NF*κ*B [62, 63], phosphatase 2A [64], ERK [65], and JNK [66] are mediated independently from PPAR-*γ*. Ideally, this issue could be answered by replicating the study in PPAR-*γ* null mouse, yet these mice are embryonically lethal. An alternative solution would be to coadminister LSN with a selective PPAR-*γ* antagonist such as GW9662 in order to elucidate PPAR-*γ* dependency [67] or use siRNA to knockdown PPAR-*γ* [68]. 

The novel non-thiazolidinedione partial PPAR-*γ* LSN may present advantages for future clinical applications as an antidiabetic treatment as well as a candidate neuroprotective agent of the dopaminergic nigrostriatal system. The finding that, in animal models of type II diabetes, LSN induced potent glucose and lipid lowering effects comparable to rosiglitazone [25], combined with our demonstration of LSN-induced neuroprotection associated with decreased neuroinflammation in mice models of PD, suggests that pure PPAR-*γ* activity is not required for metabolic or neuroprotective efficacy. Although the thiazolidinediones PPAR-*γ* agonists have greatly benefited diabetic patients and have also shown neuroprotective properties associated to anti-inflammation in animal models of PD, publications linking harmful cardiovascular effects to rosiglitazone [20–22] and bladder cancer to pioglitazone [69] have challenged their repositioning as disease modifying strategies for this neurodegenerative disorder. New reports have cleared pioglitazone of its association with oncogenesis, [70] and a clinical trial to assess its neuroprotective potential for early PD is currently ongoing (http://clinicaltrials.gov/ct2/show/NCT01280123). Furthermore, rosiglitazone's risk has recently been reevaluated [71]. Yet, concerns about the safety of both compounds linger on and make LSN or LSN-like partial or dual agonist compounds attractive alternatives.

Follow-up studies are needed to advance the characterization of LSN neuroprotective efficacy and evaluate how modulation of PPAR-*γ* and PGC1*α* affects dopaminergic nigral cell survival and how the PPAR-*α* activation contributes to its efficacy. They should include analysis of the neuroprotective effect of LSN in the peripheral nervous system, as autonomic dysfunction occurs in many PD patients. Moreover, further studies are warranted to evaluate the toxicity of potentially vulnerable peripheral organs, such as the bladder to evaluate LSN safety.

In conclusion, our results demonstrate that the novel non-thiazolidinedione PPAR-*γ* agonist LSN862 elicited neuroprotective properties associated with modulation of oxidative stress and inflammation that can be beneficial for early PD. Further studies on LSN safety and efficacy are warranted to continue towards the clinical translation of this compound.

## Supplementary Material

Supplementary Table 1 lists the primers used in real-time quantitative PCR (RT-qPCR) in striatal and nigral tissue.
Supplementary Tables 2 and 3 list effects of LSN4862 on striatal and nigral mRNA expression.
Supplementary Figure 1. LSN862 confers neuroprotection against MPTP toxicity in ARE-hPAP mice.
Supplementary Figure 2. LSN862 does not affect striatal and nigral hPAP activity in ARE-hPAP mice.

## Figures and Tables

**Table 1:** Striatal MPTP metabolism is not affected by LSN862. C57BL/6 mice received MPTP (i.p. 30 mg/kg × 5 days) or MPTP + LSN862 (p.o. 30 mg/kg). Mice were euthanized 15 or 90 minutes after the last dose of MPTP and 24 hrs after the last dose of LSN. Groups were for 15 min vehicle + MPTP (*n* = 6), 30 mg/kg + LSN + MPTP (*n* = 6); for 90 min: vehicle + MPTP (*n* = 7), 30 mg/kg LSN + MPTP (*n* = 6). No significant differences between groups were detected (two-tailed independent *t*-tests). Values represent mean ± SEM.

Measure	MPTP only (15 min)	MPTP + LSN862 (15 min)	MPTP only (90 min)	MPTP + LSN862 (90 min)
MPP^+^ level (*μ*g/g tissue)	4.14 ± 2.49	4.37 ± 0.72	3.61 ± 0.56	2.11 ± 0.20
MAO-B activity (RLU/*μ*g protein)	3760.62 ± 299.34	3320.71 ± 229.16	4421.25 ± 364.16	3824.43 ± 252.66
