# Supplementary material for: Neuroprotective Properties of a Novel Non-Thiazoledinedione Partial PPAR-γ Agonist against MPTP
Source: PPAR Res. 2013 Oct 2;2013:582809. doi: 10.1155/2013/582809 (PMC3808726; doi:10.1155/2013/582809)
Supplement: Supplementary file 1 — Supplementary Table 1 lists the primers used in real-time quantitative PCR (RT-qPCR) in striatal and nigral tissue. Supplementary Tables 2 and 3 list effects of LSN4862 on striatal and nigral mRNA expression. Supplementary Figure 1. LSN862 confers neuroprotection against MPTP toxicity in ARE-hPAP mice. Supplementary Figure 2. LSN862 does not affect striatal and nigral hPAP activity in ARE-hPAP mice. [file 582809.f1.pdf]

Supplementary Table 1. Primers used for RT-qPCR analysis of striatal and nigral tissue.

**Gene**	Forward Primer (5'-3')	Reverse Primer (5'-3')
**Dopaminergic Markers**		
TH	CTG GCC ATT GAT GTA CTG GA	ACA CAC ATG GGA AAG CCT CT
DAT	TGA ACT TTG GCC TAG CCT GT	ACA TGG AGC CAC TCA GCT CT
**Cellular Markers**		
GFAP	CGA GTC CCT AGA GCG GCA AAT G	CGG ATC TGG AGG TTG GAG AAA GTC
MAC-1	TTC TCA TGG TCA CCT CCT GC	GGT CTG ACC ATC TGA ACC TG
**Inflammatory Enzymes**		
gp91 phox	CAG GAG TTC CAA GAT GCC TG	GAT TGG CCT GAG ATT CAT CC
p67phox	CAG CCA GCT TCG GAA CAT G	GAC AGT ACC AGG ATT ACA TC
**Cytokines**		
NFkB	GCG TAC ACA TTC TGG GGA GT	ACC GAA GCA GGA GCT ATC AA
**Antioxidant Markers**		
NQO1	GCG AGA AGA GCC CTG ATT GTA CTG	TCT CAA ACC AGC CTT TCA GAA TGG
HO1	CAA GCC GAG AAT GCT GAG TTC ATG	GCA AGG GAT GAT TTC CTG CCA G
GCLM	GCC ACC AGA TTT GAC TGC CTT TG	TGC TCT TCA CGA TGA CCG AGT ACC
GCLC	ACA TCT ACC ACGC AGT CAA GG ACC	CTC AAG AAC ATC GCC TCC ATT CAG
Peroxiredoxin 3	CAC CCA GCA CGC GCC CTA TT	GGC CCA CCG GAA GGT CGT TG
Peroxiredoxin 5	GTG TCT CTC TTT GGG AAT CGT C	CTC AGA GTT GAG AGA GGA TGT TGG
SOD2	GAC TGG CAG CAT TTA GGG TT	GGA CCC AAT GAG CCA AAG AA
Thioredoxin 2	TCG CCA AGC AGC ACG GGA AG	TGA CCC CCA CAC CAG TGG CA
**Protein Kinases**		
AMPK	GGG GAC ACG CTT GGT GTC GG	GCT GGA ACA GAC GGC GGC TT
MAPK	CCT CAA GCC TGA AAA TCT GC	GTC TCT GCG CTT CCA GTA CC
**PPAR-γ markers**		
PPAR-γ	CCA GAG TCT GCT GAT CTG CG	GCC ACC TCT TTG CTC TGA TC
PGC1-α	TGA TGT GAA TGA CTT GGC TAC AGA CA	GCT CAT TGT TGT ACT GGT TGG ATA TG
**Housekeeping gene**		
Beta-actin	CAT GAA GAT CCT GAC CGA GCG TG	TCT GCT GGA AGG TGG ACA GTG AGG

Supplementary Table 2. Effect of LSN862 (LSN) on striatal mRNA expression. Striatal tissue was collected at 24 hrs, 72 hrs, and 21 days after the last dose of MPTP and 24 hrs after LSN862. The groups were vehicle + PBS (24 hrs: n = 5; 72 hrs: n = 4; 21 days: n = 4); vehicle + MPTP (24 hrs: n = 5; 72 hrs: n = 5; 21 days: n= 6); 30 mg/kg LSN + PBS (24 hrs: n = 4; 72 hrs: n = 3; 21 days n = 4; 30 mg/kg LSN + MPTP (24 hrs: n = 5; 72 hrs n = 4; 21 days n = 5). All data are standardized to β-actin presented as mean ± SEM compared to vehicle + PBS controls. Two-Way ANOVA with Repeated Measures: [a]Vehicle + PBS vs. Vehicle + MPTP, $P < 0.05$; [b]Vehicle + MPTP vs. 30mg/kg LSN + MPTP, $P < 0.05$; [c]Vehicle + PBS vs. 30mg/kg LSN + PBS, $P < 0.05$.

	**Vehicle + PBS**			**Vehicle + MPTP**			**30 mg/kg LSN + PBS**			**30 mg/kg LSN + MPTP**		
**Gene**	24 hrs	72 hrs	21 days	24 hrs	72 hrs	21 days	24 hrs	72 hrs	21 days	24 hrs	72 hrs	21 days
**Dopaminergic markers**												
TH	1.00 ± 0.34	1.00 ± 0.58	1.00 ± 0.09	0.37 ± 0.19	0.09 ± 0.06	0.39 ± 0.08	0.98 ± 0.57	0.98 ± 0.57	1.30 ± 0.36	0.20 ± 0.06	0.52 ± 0.01	1.15 ± 0.51
DAT	1.00 ± 0.49	1.00 ± 0.65	1.00 ± 0.39	0.49 ± 0.21	0.004 ± 0.003	0.27 ± 0.10	0.97 ± 0.36	0.88 ± 0.01	1.41 ± 0.73	0.45 ± 0.15	0.30 ± 0.11	1.20 ± 0.59
**Cellular Markers**												
GFAP	1.00 ± 0.40	1.00 ± 0.35	1.00 ± 0.21	**7.38 ± 0.98**[a,b]	**2.49 ± 1.16**[a]	0.95 ± 0.26	0.43 ± 0.03	0.71 ± 0.67	0.45 ± 0.15	**0.92 ± 0.22**[b]	0.77 ± 0.46	0.51 ± 0.14
MAC-1	1.00 ± 0.42	1.00 ± 0.42	1.00 ± 0.17	**4.13 ± 1.26**[a,b]	4.92 ± 2.83	0.83 ± 0.23	0.85 ± 0.21	0.70 ± 0.04	0.94 ± 0.35	**1.55 ± 0.56**[b]	0.23 ± 0.18	0.99 ± 0.15
**Inflammatory Enzymes**												
gp91phox	1.00 ± 0.58	1.00 ± 0.48	1.00 ± 0.44	**2.5 ± 0.39**[a,b]	0.31 ± 0.17	0.10 ± 0.03	0.95 ± 0.62	0.10 ± 0.08	0.07 ± 0.01	**1.14 ± 0.38**[b]	0.15 ± 0.08	0.10 ± 0.03
p67phox	1.00 ± 0.37	1.00 ± 0.32	1.00 ± 0.36	1.40 ± 0.58	0.02 ± 0.01	**37.48 ± 11.47**[a,b]	0.86 ± 0.36	0.05 ± 0.003	2.46 ± 0.47	0.02 ± 0.01	0.47 ± 0.44	**2.74 ± 1.31**[b]
NFkB	1.00 ± 0.16	1.00 ± 0.59	1.00 ± 0.15	**5.80 ± 3.45**[a]	0.03 ± 0.02	0.18 ± 0.04	2.02 ± 1.33	0.71 ± 0.25	0.19 ± 0.05	3.43 ± 1.01	0.33 ± 0.16	0.14 ± 0.04
**Antioxidant Markers**												
NQO1	1.00 ± 0.37	1.00 ± 0.43	1.00 ± 0.18	**5.00 ± 1.94**[a,b]	0.51 ± 0.25	0.12 ± 0.02	0.91 ± 0.27	0.85 ± 0.79	0.34 ± 0.12	**2.31 ± 0.72**[b]	1.46 ± 0.63	0.11 ± 0.02
HO-1	1.00 ± 0.11	1.00 ± 0.49	1.00 ± 0.48	2.27 ± 0.20	0.18 ± 0.09	2.57 ± 0.84	1.11 ± 0.25	0.61 ± 0.43	1.81 ± 1.49	0.92 ± 0.35	1.23 ± 0.44	2.69 ± 0.92
GCLM	1.00 ± 0.34	1.00 ± 0.45	1.00 ± 0.46	1.55 ± 0.46	**5.80 ± 2.82**[a]	1.02 ± 0.31	0.56 ± 0.21	1.32 ± 0.66	1.16 ± 0.32	0.96 ± 0.30	4.07 ± 1.79	1.24 ± 0.40
GCLC	1.00 ± 0.38	1.00 ± 0.41	1.00 ± 0.09	3.75 ± 1.23	**12.92 ± 3.65**[a]	0.15 ± 0.07	2.01 ± 0.92	0.87 ± 0.26	0.08 ± 0.02	1.69 ± 0.81	15.47 ± 3.32	0.03 ± 0.01
Peroxiredoxin 5	1.00 ± 0.47	1.00 ± 0.35	1.00 ± 0.36	0.31 ± 0.12	1.24 ± 0.62	0.58 ± 0.15	0.58 ± 0.16	0.83 ± 0.17	0.70 ± 0.23	0.45 ± 0.39	0.74 ± 0.40	0.41 ± 0.08
SOD2	1.00 ± 0.51	1.00 ± 0.17	1.00 ± 0.32	0.45 ± 0.14	0.05 ± 0.02	0.44 ± 0.14	0.16 ± 0.05	1.34 ± 0.91	0.28 ± 0.06	0.88 ± 0.38	0.51 ± 0.20	0.35 ± 0.09
Thioredoxin 2	1.00 ± 0.54	1.00 ± 0.51	1.00 ± 0.40	2.55 ± 0.56	0.78 ± 0.30	0.62 ± 0.21	0.72 ± 0.21	0.06 ± 0.01	0.51 ± 0.18	1.14 ± 0.49	0.42 ± 0.41	0.22 ± 0.05
**Protein Kinases**												
AMPK	1.00 ± 0.16	1.00 ± 0.47	1.00 ± 0.36	**3.41 ± 0.56**[a,b]	**0.68 ± 0.20**[b]	0.06 ± 0.02	**2.79 ± 1.03**[c]	1.18 ± 0.69	0.62 ± 0.27	**1.82 ± 0.66**[b]	**3.73 ± 0.85**[b]	0.13 ± 0.03
MAPK	1.00 ± 0.58	1.00 ± 0.08	1.00 ± 0.35	0.05 ± 0.02	0.03 ± 0.01	0.41 ± 0.09	**13.31 ± 8.74**[c]	1.69 ± 1.19	5.36 ± 4.92	4.34 ± 2.64	3.15 ± 0.90	4.93 ± 2.23
**PPAR-γ markers**												
PPAR-γ	1.00 ± 0.35	1.00 ± 0.17	1.00 ± 0.31	**2.04 ± 0.25**[a,b]	**0.30 ±0.12**[a]	0.39 ± 0.08	1.39 ± 0.33	**7.01 ± 1.5**[c]	0.91 ± 0.31	**0.63 ± 0.25**[b]	3.56 ± 0.64	1.09 ± 0.32
PGC1-α	1.00 ± 0.42	1.00 ± 0.57	1.00 ± 0.49	**22.65 ±0.57**[a,b]	0.10 ± 0.42	0.31 ± 0.07	0.35 ± 0.23	0.37± 0.23	0.58 ± 0.07	**0.44 ± 0.19** [b]	0.61± 0.20	0.43 ± 0.13

Supplementary Table 3. Effect of LSN862 (LSN) on nigral mRNA expression. Substantia nigra tissue was collected at 24 hrs, 72 hrs, and 21 days after the last dose of MPTP and 24 hrs after LSN862 . The groups were vehicle + PBS (24 hrs: n = 5; 72 hrs: n = 4; 21 days: n = 4); vehicle + MPTP (24 hrs: n = 5; 72 hrs: n = 5; 21 days: n= 6); 30 mg/kg LSN + PBS (24 hrs: n = 4; 72 hrs: n = 3; 21 days n = 4; 30 mg/kg LSN + MPTP (24 hrs: n = 5; 72 hrs n = 4; 21 days n = 5). All data are standardized to β-actin presented as mean ± SEM compared to vehicle + PBS controls. Two-Way ANOVA with Repeated Measuress: [a]Vehicle + PBS vs. Vehicle + MPTP, *P* < 0.05; [b]Vehicle + MPTP vs. 30mg/kg LSN + MPTP, *P* < 0.05; [c]Vehicle + PBS vs. 30mg/kg LSN + PBS, *P* < 0.05.

	**Vehicle + PBS**			**Vehicle + MPTP**			**30 mg/kg LSN + PBS**			**30 mg/kg LSN + MPTP**		
**Gene**	24 hrs	72 hrs	21 days	24 hrs	72 hrs	21 days	24 hrs	72 hrs	21 days	24 hrs	72 hrs	21 days
**Dopaminergic markers**												
TH	1.00 ± 0.53	1.00 ± 0.35	1.00 ± 0.45	0.06 ± 0.04	0.02 ± 0.01	0.17 ± 0.04	1.27 ± 0.75	0.86 ± 0.33	1.33 ± 0.53	0.58 ± 0.32	0.15 ± 0.04	0.47 ± 0.34
DAT	1.00 ± 0.66	1.00 ± 0.62	1.00 ± 0.41	0.06 ± 0.05	0.62 ± 0.26	0.12 ± 0.04	1.45 ± 0.77	1.15 ± 0.32	1.33 ± 0.74	0.18 ± 0.09	0.58 ± 0.22	0.32 ± 0.05
**Cellular Markers**												
GFAP	1.00 ± 0.29	1.00 ± 0.47	1.00 ± 0.40	**262.7 ± 76.92**[a,b]	3.84 ± 1.39	1.39 ± 0.65	32.0 ± 6.96	0.04 ± 0.02	0.90 ± 0.72	**18.96 ± 10.82**[,b]	0.15 ± 0.09	1.58 ± 0.63
MAC-1	1.00 ± 0.35	1.00 ± 0.33	1.00 ± 0.10	**5.16 ± 2.43**[a,b]	**3.24 ± 0.46**[a,b]	2.32 ± 0.72	1.47 ± 0.62	0.03 ± 0.01	1.34 ± 0.32	**1.38 ± 0.91**[b]	**0.80 ± 0.53**[b]	2.06 ± 0.80
**Inflammatory Enzymes**												
gp91phox	1.00 ± 0.46	1.00 ± 0.30	1.00 ± 0.62	**7.12 ± 3.11**[a,b]	0.74 ± 0.32	1.18 ± 0.52	1.03 ± 0.56	0.20 ± 0.18	3.15 ± 0.81	**1.63 ± 0.92**[b]	0.89 ± 0.44	1.02 ± 0.24
p67phox	1.00 ± 0.32	1.00 ± 0.29	N/A	**4.47 ± 1.99**[b]	**2.75 ± 1.20**[a,b]	N/A	0.70 ± 0.39	0.30 ± 0.12	N/A	**0.09 ± 0.03**[b]	**0.94 ± 0.79**[b]	N/A
NFkB	1.00 ± 0.35	1.00 ± 0.14	1.00 ± 0.47	3.29 ± 1.20	0.54 ± 0.24	1.05 ± 0.39	0.37 ± 0.17	0.15 ± 0.13	0.94 ± 0.22	2.40 ± 0.73	0.02 ± 0.01	0.62 ± 0.22
**Antioxidant Markers**												
NQO1	1.00 ± 0.38	1.00 ± 0.22	1.00 ± 0.34	**5.66 ± 1.86**[a]	0.88 ± 0.32	0.68 ± 0.10	0.98 ± 0.46	0.08 ± 0.05	1.13 ± 0.38	4.29 ± 1.81	0.34 ± 0.14	0.33 ± 0.10
HO-1	1.00 ± 0.24	1.00 ± 0.10	1.00 ± 0.62	1.01 ± 0.20	0.35 ± 0.13	1.63 ± 0.47	0.51 ± 0.12	0.67 ± 0.25	8.35 ± 4.99	2.47 ± 1.15	0.38 ± 0.06	0.82 ± 0.29
GCLM	1.00 ± 0.21	1.00 ± 0.40	1.00 ± 0.04	1.72 ± 0.25	**3.06 ± 0.53**[a]	0.01 ± 0.002	0.78 ± 0.38	0.99 ± 0.42	0.001± 0.0005	1.43 ± 0.50	2.47 ± 0.76	0.003 ± 0.0009
GCLC	1.00 ± 0.28	1.00 ± 0.30	1.00 ± 0.07	**3.42 ± 0.96**[a]	**3.09 ± 1.10**[a]	0.06 ± 0.004	0.27 ± 0.16	0.31 ± 0.14	0.02 ± 0.01	1.94 ± 0.85	6.11 ± 1.85	0.02 ± 0.006
Peroxiredoxin 5	1.00 ± 0.29	1.00 ± 0.36	1.00 ± 0.19	2.23 ± 0.61	0.80 ± 0.19	0.96 ± 0.26	6.31 ± 3.76	0.24 ± 0.21	0.72 ± 0.06	3.24 ± 1.31	0.64 ± 0.45	0.89 ± 0.31
SOD2	1.00 ± 0.35	1.00 ± 0.39	1.00 ± 0.42	0.74 ± 0.21	0.23 ± 0.15	**2.53 ± 0.38**[a,b]	0.09 ± 0.04	0.03 ± 0.01	1.29 ± 0.38	1.04 ± 0.38	0.07± 0.04	**1.14 ± 0.31**[b]
Thioredoxin 2	1.00 ± 0.43	1.00 ± 0.15	1.00 ± 0.40	0.62 ± 0.09	3.09 ± 1.49	0.62 ± 0.22	0.40 ± 0.21	0.29 ± 0.15	0.51 ± 0.19	0.72 ± 0.29	0.13 ± 0.04	0.22± 0.05
**Protein Kinases**												
AMPK	1.00 ± 0.41	1.00 ± 0.39	1.00 ± 0.27	1.93 ± 0.02	3.40 ± 2.04	0.37 ± 0.13	4.40 ± 2.53	0.91 ± 0.33	0.37 ± 0.09	13.12 ± 8.55	0.76 ± 0.15	0.22 ± 0.05
MAPK	1.00 ± 0.27	1.00 ± 0.66	1.00 ± 0.19	0.04 ± 0.01	0.17 ± 0.08	1.01 ± 0.18	0.51 ± 0.15	2.98 ± 1.81	0.62 ± 0.05	2.67 ± 1.39	1.92 ± 0.76	0.37 ± 0.05
**PPAR-γ markers**												
PPAR-γ	1.00 ± 0.15	1.00 ± 0.43	1.00 ± 0.47	0.87 ± 0.28	0.02 ± 0.01	1.90 ± 1.14	0.37 ± 0.18	4.95 ± 1.67	0.71 ± 0.17	0.90 ± 0.54	1.89 ± 0.5	0.99 ± 0.30
PGC1-a	1.00 ± 0.14	1.00 ± 0.33	1.00 ± 0.25	7.25 ± 4.12	0.19 ± 0.06	0.57 ± 0.12	0.79 ± 0.23	0.12 ± 0.06	0.85 ± 0.22	2.93 ± 1.32	0.11 ± 0.01	0.43 ± 0.15

Supplementary Figure Legends

Supplementary Figure 1. LSN862 confers neuroprotection against MPTP toxicity in ARE-hPAP mice. Striata from ARE-hPAP mice were harvested 7 days after the last dose of MPTP and 24 hrs after treatment with LSN. A) Western blot of TH protein levels. B) Densitometric analysis of TH western blot relative to ß-actin (means ± SEM). Groups: vehicle + PBS (n = 7), vehicle + MPTP (n = 7), 30 mg/kg LSN + PBS (n = 8), 30 mg/kg LSN + MPTP (n = 6)). # *P* = 0.029 (vehicle + PBS vs. vehicle + MPTP), * *P* = 0.05, (vehicle + MPTP vs. 30 mg/kg LSN + MPTP). HPLC analysis of striatal catecholamine levels vehicle + PBS (n = 8), vehicle + MPTP (n = 9), 30 mg/kg LSN + PBS (n = 9), 30 mg/kg LSN + MPTP (n = 7), C) Dopamine: # *P* = 0.003 (vehicle + PBS vs. vehicle + MPTP). D) DOPAC: # *P* = 0.022 (vehicle + PBS vs. vehicle + MPTP). E) HVA: # *P* = 0.014 (vehicle + PBS vs. vehicle + MPTP), F) HVA:DA ratio.

Supplementary Figure 2. LSN862 does not affect striatal and nigral hPAP activity in ARE-hPAP mice. Groups: vehicle + PBS (n = 5), vehicle + MPTP (n = 5), 30 mg/kg LSN + PBS (n = 3), 30 mg/kg LSN + MPTP (n = 5). A) Striatal hPAP activity levels (means ± SEM). # *P* = 0.040 (vehicle + PBS vs. vehicle + MPTP). B) Nigral hPAP activity levels; # *P* = 0.031 (vehicle + PBS vs. vehicle + MPTP).

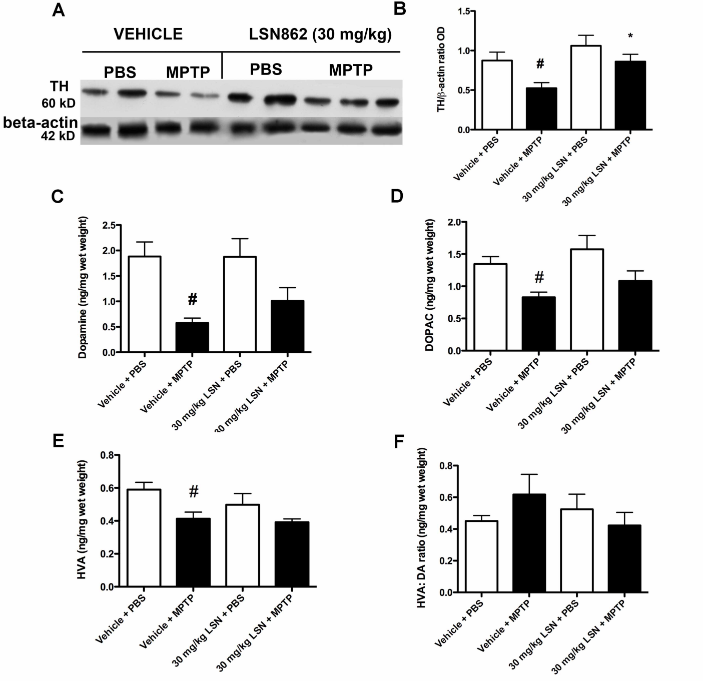
A
VEHICLE
LSN862 (30 mg/kg)
PBS
MPTP
PBS
MPTP
TH
60 kD
beta-actin
42 kD
B
TH/β-actin ratio OD
Vehicle + PBS
Vehicle + MPTP
30 mg/kg LSN + PBS
30 mg/kg LSN + MPTP
C
Dopamine (ng/mg wet weight)
D
DOPAC (ng/mg wet weight)
E
HVA (ng/mg wet weight)
F
HVA: DA ratio (ng/mg wet weight)

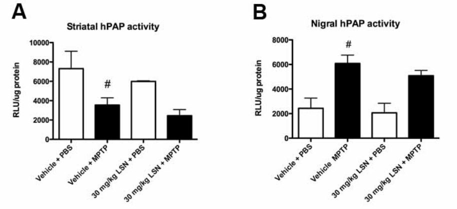
A
Striatal hPAP activity
RLU/ug protein
10000
8000
6000
4000
2000
0
#
Vehicle + PBS
Vehicle + MPTP
30 mg/kg LSN + PBS
30 mg/kg LSN + MPTP
B
Nigral hPAP activity
RLU/ug protein
8000
6000
4000
2000
0
#
Vehicle + PBS
Vehicle MPTP
30 mg/kg LSN + PBS
30 mg/kg LSN + MPTP
